# FOLFIRINOX-R study design: a phase I/II trial of FOLFIRINOX plus regorafenib as first line therapy in patients with unresectable RAS-mutated metastatic colorectal cancer

**DOI:** 10.1186/s12885-021-08312-7

**Published:** 2021-05-17

**Authors:** Antoine Adenis, Thibault Mazard, Julien Fraisse, Patrick Chalbos, Brice Pastor, Ludovic Evesque, Francois Ghiringhelli, Caroline Mollevi, Stéphanie Delaine, Marc Ychou

**Affiliations:** 1 IRCM, Inserm, Université Montpellier, ICM, Montpellier, France; 2 Department of Medical Oncology, Montpellier Cancer Institute (ICM), Montpellier, France; 3 Department of Gastrointestinal Oncology, Institut Régional du Cancer de Montpellier, 208 Avenue des Apothicaires, 34000 Montpellier, France; 4 Biometrics Unit, Montpellier Cancer Institute (ICM), Montpellier, France; 5 Department of Clinical Research, Montpellier Cancer Institute (ICM), University of Montpellier, Montpellier, France; 6 IRCM, Inserm U1194, Montpellier, France; 7 Department of Medical Oncology, Centre Antoine Lacassagne, Nice, France; 8 Department of Medical Oncology, Georges François Leclerc, Dijon, France

**Keywords:** Colorectal cancer, Chemotherapy triplet, Regorafenib, Phase 1–2 trial

## Abstract

**Background:**

The chemotherapy triplet FOLFOXIRI combined to the anti-VEGF antibody bevacizumab is an option in selected patients with metastatic colorectal cancer. In this setting, RAS-mutated metastatic colorectal cancer do not benefit the same from treatment than RAS-wildtype metastatic colorectal cancer do. Together with its antiangiogenic properties, the tyrosine-kinase inhibitor regorafenib has also anti-proliferative activities whatever the RAS status is. The present trial aims at studying the safety and the efficacy of regorafenib in combination with FOLFIRINOX – a chemotherapy triplet using a different dosing schedule than FOLFOXIRI - in patients with *RAS*-mutated metastatic colorectal cancer.

**Methods:**

FOLFIRINOX-R is a prospective, multicentric, non-randomised, dose-finding phase 1–2 trial. The primary endpoints are the determination of the maximum tolerated dose, the recommended phase 2 dose, and the proportion of patients achieving disease control at 48-weeks. Phase 1 follows a 3 + 3 design (12 to 24 patients to be included). Sixty nine patients will be necessary in phase 2, including 5% non-evaluable ones, with the following assumptions, one-stage Fleming design, α = 5%, β = 20%, p0 = 35% and p1 = 50%. Key eligibility criteria include Eastern Cooperative Oncology Group Performance Status of ≤1 and RAS-mutated metastatic colorectal cancer not amenable to surgery with curative intent and not previously treated for metastatic disease. FOLFIRINOX (oxaliplatin 85 mg/m^2^, folinic acid 400 mg/m^2^, irinotecan 150–180 mg/m^2^, 5-fluorouracil: 400 mg/m^2^ then 2400 mg/m^2^ over 46 h) is administered every 14 days. Regorafenib (80 to 160 mg, as per dose-level) is administered orally, once daily on days 4 to 10 of each cycle.

**Discussion:**

FOLFIRINOX-R is the first phase I/II study to evaluate the safety and efficacy of regorafenib in combination with FOLFIRINOX as frontline therapy for patients with RAS-mutated metastatic colorectal cancer.

**Trial registration:**

EudraCT: 2018-003541-42; ClinicalTrials.gov: NCT03828799.

## Background

### Metastatic colorectal cancer

Colorectal cancer (CRC) is a major cause of morbidity and mortality globally [1]. More than 50% of patients will develop metastatic disease, and while some patients with technically resectable liver (or lung) metastases could be cured with surgery only, most of the patients will require palliative systemic therapy as their metastases are found unresectable [2]. As per European Society for Medical Oncology guidelines, frontline chemotherapy for metastatic CRC (mCRC) commonly involves the doublet (2-CTx) regimens of 5-fluorouracil, folinic acid, and either oxaliplatin (FOLFOX) or irinotecan (FOLFIRI) [2]. However, Falcone et al. [3] reported higher response rates and better survival rates with the use of a chemotherapy triplet (3-CTx) that combined 5-fluorouracil, folinic acid, oxaliplatin and irinotecan (FOLFOXIRI) over 2-CTx. Similarly, our group reported favorable outcomes in patients with unresectable liver metastases from colorectal origin, with the same three drugs but using a different dosing schedule, i.e. the FOLFIRINOX regimen [4]. It is also accepted that the addition of targeted therapies such as bevacizumab (a monoclonal antibody which binds circulating vascular endothelial growth factor-A) or as monoclonal antibodies (cetuximab, panitumumab) directed to epidermal growth factor receptor to 2-CTx or 3-CTx it is beneficial in fit patients when tumor shrinkage is a major objective to allow conversion surgery [2].

*RAS* mutations are identified in about 60% of mCRC tumors and are known as negative predictors of efficacy for anti-epidermal growth factor receptor therapy in mCRC [5]. *RAS* status also carries distinct prognostic information, as reported in a series of 1239 mCRC patients who have been treated with 2-CTx in five randomized trials [5]. Interestingly, the poor prognosis related to *RAS* mutations was observed across different treatment regimens (subgroups of irinotecan- and oxaliplatin-treated patients as well as in bevacizumab- and non-bevacizumab-treated patients). Median progression-free survival (PFS) and overall survival (OS) were 10.3 vs. 9.5 months and 26.9 vs. 21.1 months in *RAS*-wild type (and *BRAF*-wild type) and *RAS*-mutated tumors, respectively [5]. Similar survival differences by *RAS* status were also observed in clinical trials which addressed the benefit of 3-CTx over 2-CTx. The TRIBE consortium reported that 3-CTx (FOLFOXIRI) combined with bevacizumab provided a significantly longer PFS and OS than did 2-CTx with FOLFIRI plus bevacizumab [6, 7]. Actually, when looking at survival by *RAS* status, Cremolini et al. [7] reported that median OS was 37.1 months (95%CI, 29.7–42.7) in the *RAS*- and *BRAF*-wild-type subgroup compared with 25.6 months (95%CI, 22.4–28.6) in the *RAS*-mutated subgroup (HR 1.49, 95%CI, 1.11–1·99). For sure, we need to increase the treatment benefit in this setting of RAS-mutated mCRC.

### Regorafenib

Regorafenib is a small-molecule inhibitor of multiple membrane-bound and intracellular kinases such as *RET, VEGFR-1, − 2, − 3, c-KIT, PDGFR, FGFR1, TIE2, DDR1, Trk2A, Eph2A, SAPK2, PTK5, Abl, CSF1R, RAF-1, BRAF*, and *BRAF V600E* [8] which is approved for refractory mCRC. Beyond its well-known antiangiogenic properties, regorafenib has also less-known anti-proliferative activities in human colon cancer cell lines [8]. Interestingly, regorafenib potently inhibits growth of patient-derived CRC xenografts alone and in combination with irinotecan [8–10]. Two phase III trials demonstrated an overall survival benefit for regorafenib (160 mg orally, once daily for the first 21 days of each 28-day cycles) over placebo in patients with mCRC who progressed on standard therapies, whatever the *RAS* status was [11, 12]. However, half of the patients who received regorafenib in that setting progressed 2 months after the onset of treatment [11, 12] and were then possibly unnecessary exposed to adverse events (AEs). Unfortunately, there is no fully accepted biomarker able to predict regorafenib benefit. Two phase II trials also studied the safety and efficacy profile of regorafenib when combined to chemotherapy in patients with mCRC [13, 14]. The study from Schultheis et al. [13] was designed to explore whether the addition of regorafenib to FOLFOX or FOLFIRI could be feasible as first- or second-line treatment of mCRC. Forty-five patients were treated every 2 weeks with 5-fluorouracil 400 mg/m^2^ bolus then 2400 mg/m^2^ over 46 h, folinic acid 400 mg/m^2^, and either oxaliplatin 85 mg/m^2^ or irinotecan 180 mg/m^2^. On days 4 to 10, patients received regorafenib 160 mg orally once daily. Regorafenib showed acceptable tolerability in combination with chemotherapy. The most frequent grade 3–4 AEs were: neutropenia (45%), Hand-Foot Skin Reaction (15%), diarrhea (10%), and hypophosphatemia (12%). The study from Sanoff et al. [14] was designed to show whether the addition of regorafenib to FOLFIRI (same regimen than in the Schultheis trial [13]) improves PFS (over a placebo-FOLFIRI arm) when given as second-line treatment for patients previously treated with oxaliplatin and fluoropyrimidine-based regimen. The study met its primary endpoint, demonstrating that the addition of regorafenib to FOLFIRI prolongs PFS compared to FOLFIRI alone with a HR of 0.72. When looking at tumor response, the authors found that regorafenib (combined to chemotherapy) provided more partial responses than chemotherapy plus placebo (35% vs. 19%, *p* = 0.045). Tolerability was acceptable, with little increase in toxicity compared to the control chemotherapy regimen [14]. Based on these encouraging results, it could be worth studying the combination of 3-CTx to regorafenib, with the ultimate objective of providing a more efficacious alternative to a selected population of eligible patients.

### Aim of the study

The present FOLFIRINOX-R trial aims at studying the safety, tolerability and efficacy of regorafenib in combination with FOLFIRINOX in patients with *RAS*-mutated mCRC.

## Methods and design

### Study design and treatment

FOLFIRINOX-R trial is a standard 3 + 3 design for dose escalation/de-escalation followed by a phase II trial when the recommended phase II dose (RP2D) is determined. It is designed as a single-arm, prospective, non-randomized, open label, multicenter, dose-finding phase I/II trial. It aims at determining the maximum tolerated dose (MTD) and the RP2D of the combination of regorafenib and FOLFIRINOX in the phase I-part of the trial. It aims too at evaluating the efficacy of the treatment in the phase II - part of the trial.

**Fig. 1:**
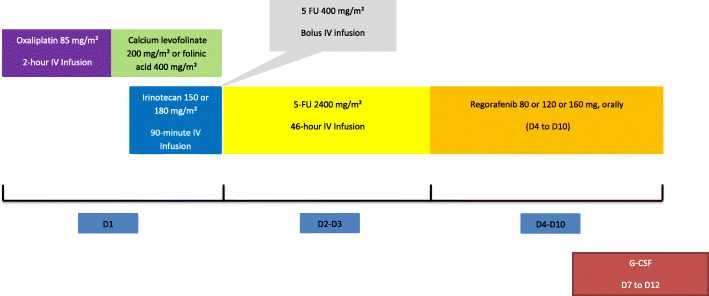
Treatment design

FOLFIRINOX is administered as per standard procedures every 14 days (1 cycle = 14 days) as follows, oxaliplatin 85 mg/m^2^ on day 1, IV infusion over 2 h, immediately followed by folinic acid 400 mg/m^2^ or calcium levofolinate 200 mg/m^2^ given as a 2-h IV infusion, with the addition of irinotecan 150–180 mg/m^2^ as per dose-level given as a 90-min intravenous infusion through a Y-connector immediately followed by 5-fluorouracil: 400 mg/m^2^ IV bolus then 2400 mg/m^2^ over 46 h continuous infusion. Primary prophylactic G-CSF is delivered from Day-7 to Day-12. Regorafenib is administered orally at a dose of 80 to 160 mg, as per dose-level, once daily on days 4 to 10 of each cycle (see Fig. 1 for an overview of the study design).

The pre-defined dose levels are the following, step − 1 (0 to 6 patients to be treated): irinotecan 150 mg/m^2^ plus regorafenib 80 mg/m^2^; step 1 (3 to 6 patients to be treated): irinotecan 150 mg/m^2^ plus regorafenib 120 mg/m^2^; step 2 (3 to 6 patients to be treated): irinotecan 180 mg/m^2^ plus regorafenib 120 mg/m^2^; step 3 (3 to 6 patients to be treated): irinotecan 180 mg/m^2^ plus regorafenib 160 mg/m^2^.

Twelve cycles of FOLFIRINOX have to be delivered consecutively or not. Indeed, treatment can be temporarily stopped to perform surgical resection or regional procedures (radiofrequency, cryoablation, radiation therapy) if the disease becomes accessible to them but if it happens, before the end of the 12 planned cycles, the treatment has to be resumed within 4 to 8 weeks after the loco-regional procedure completion in order to reach a total number of 12 cycles delivered. Regorafenib dosing is 80 to 160 mg/m^2^ according to each level of dose, by mouth daily on days 4 to 10 of each 14 days cycle, according with the regorafenib schedule reported by Schultheis et al. [13]. Regorafenib is continued until tumor progression or unacceptable toxicity, even after 12 cycles of FOLFIRINOX. Clinical evaluation will be made at each cycle. The following biologic assessments are performed before each cycle: RBC, hemoglobin, hematocrit, platelet count, WBC, coagulation panel (Prothrombin time, Partial Thromboplastin Time), electrolyte panel (sodium, potassium, chloride, magnesium, calcium, bicarbonates, phosphate), chemistry panel (albumin, lipase, glucose, serum creatinine, MDRD clearance, uric acid, urea, protein total), hepatic panel (AST, ALT, direct and indirect bilirubin, alkaline phosphatase, gammaglutamyl transpeptidase, lactate dehydrogenase), urine analysis (PH, protein, glucose, bilirubin, ketones, blood cells and leukocytes). TSH is performed every 6 weeks. The following biologic assessments is performed on day 8 of cycle 1, 2, 3: RBC, hemoglobin, hematocrit, platelet count and WBC (including differential neutrophil, lymphocyte, monocyte, basophil and eosinophil counts), chemistry panel (albumin, total protein, lipase, glucose, serum creatinine, MDRD clearance, uric acid and Urea), liver function panel (AST, ALT, direct and indirect bilirubin, gammaglutamyl transpeptidase, lactate dehydrogenase). Blood pressure is monitored every other week for the three first cycles of study treatment. Clinical, morphological imaging (RECIST criteria, v1.1) and biological (CEA and CA19.9) tumor assessment are done every 8 weeks. Prespecified dose adjustments (dose modifications and/or dose delay) for regorafenib or FOLFIRINOX could be made to manage adverse events.

Recruitment is currently active in three cancer centers (Dijon, Montpellier, and Nice) in France. Written informed consent is obtained from each patient before any screening and inclusion procedure. Patients remain on study until one of the following condition applies, study withdrawal, discontinuation of treatment or death.

### Study objectives and endpoints

The primary objective of the phase 1 – part of the study is to determine the MTD and the RP2D of the combination of regorafenib and FOLFIRINOX. DLT is defined as the occurrence of one or more of the following toxicities during the three first cycles of treatment:

- Unplanned interruption > 7 days of regorafenib due to drug-related toxicity
- Grade (gr.) ≥ 3 (CTCAE v5) non-hematologic toxicity, except: gr. 3 nausea, gr. 3 vomiting, gr. 3 diarrhea, and gr. ≥ 3 lipase elevation without signs of pancreatitis,
- Gr. ≥ 2 posterior reversible encephalopathy syndrome,
- Gr. ≥ 2 retinopathy,
- Any of the following liver-specific DLTs: gr. ≥ 3 bilirubin increase, gr. ≥ 3 AST and/or ALT increase, or AST and/or ALT increase > 3 x UNL with concurrent bilirubin increase (> 2 x UNL)
- Gr. 4 neutropenia lasting > 3 days,
- Gr. ≥3 febrile neutropenia (ANC < 1000/mm3 with fever ≥38.5 °C),
- Gr. 4 anemia,
- Platelets < 25,000 /mm3 or platelets < 50,000 /mm3 with bleeding,
- INR or PTT elevation of Gr. ≥ 3 with bleeding,
- Gr. ≥ 3 hemorrhage/bleeding events.

The primary objective of the Phase 2 – part of the study is to evaluate the efficacy, with the 48-week disease-control rate assessment. The latter is defined as the rate of non-progressing patients (after central review) at 48-week post-initiation of therapy, in all treated patients.

The secondary endpoints of the phase 2 – part of the project include safety (according to NCI-CTC v5 scale), overall response rate (according to RECIST v1.1), duration of response, 8-week response rate, deepness of response, disease-control rate, resectability (R0/R1) rate among patients with liver-only metastases, progression-free survival, progression-free survival since maintenance therapy onset with regorafenib, and the overall survival.

### Statistical design

A minimum of 12 and a maximum of 24 patients will be included in the phase I – part of the study, with a minimum of 3 and a maximum of 6 patients per dose level. Six patients will be included at the RP2D. It would be necessary to include 65 evaluable patients (actually 69 patients, including 5% non-evaluable patients) in the phase II-part of the trial with the following assumptions, one-stage Fleming design, α = 5%, β = 20%, p0 (the probability of maximal inefficiency) = 35% and p1 (the probability of minimal efficiency) = 50%. FOLFIRINOX-R will be considered sufficiently effective (rejection of the null hypothesis) if at least 29 successes occur (defined as a 48-week disease control) out of 65 evaluable patients, the success rate (48-weeks disease control rate) being significantly greater than 35%. FOLFIRINOX-R will be considered insufficiently effective (rejection of the alternative hypothesis) in case of 28 or less successes out of 65 evaluable patients, the success rate (48-weeks disease control rate) being significantly lower than 50%. Overall, a maximum of 87 patients (18 patients for phase I + 69 patients for phase II, including 6 patients treated at the RP2D during the phase I) will be included.

### Study procedures

**Fig. 2:**
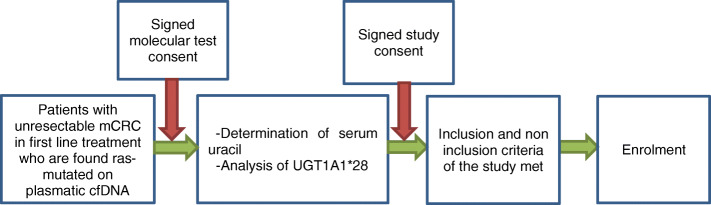
Selection procedure

Enrollment into FOLFIRINOX-R will be performed in two stages (a molecular testing then a full testing if appropriate) (Fig. 2) with two separate inform-consent forms. Only patients with the following characteristics, histological or cytological documented metastatic colorectal cancer not amenable to surgical resection with curative intent, no prior therapy for metastatic disease, measurable disease, aged 18 or older, with an Eastern Cooperative Oncology Group performance status ≤1 and a life expectancy of at least 3 months, will undergo the molecular testing which includes a circulating cell-free DNA (ccfDNA) *RAS*-mutation detection, a serum uracil assessment, and an assessment of the polymorphisms of UGT1A*28.

### Study population

The patients identified with all the conditions or characteristics listed Table 1 could be included in the study, whereas those identified with at least one condition or characteristic listed Table 2 could not be included.

**Table 1:** Inclusion criteria

- Have histological or cytological documentation of adenocarcinoma of the colon or rectum.	
- Have synchronous or metachronous metastatic colorectal cancer not amenable to surgical resection with curative intent and no prior therapy for metastatic disease.	
- Have a tumor RAS mutation	
- Have a wild-type homozygous or an heterozygous status of the UGT1A1 regarding UGT1A*28.	
- Have a serum uracil < 16 ng/ml	
- Have a measurable disease, according to RECIST version 1.1	
- Have signed written informed consent	
- Be aged 18 or older	
- Have an Eastern Cooperative Oncology Group performance status ≤1 and a life expectancy of at least 3 months.	
- Have an adequate bone marrow, renal and liver functions as evidenced by the following laboratory requirements: absolute neutrophil count ≥1500/ mm3, platelet count ≥100,000/mm3, Hemoglobin ≥9 g/dL, serum creatinine ≤1.5 x upper limit of normal (ULN), serum calcium ≥ LLN and ≤ 1.2 x UNL; serum magnesium ≥ LLN and ≤ 1.2 x UNL; Kalemia ≥ LLN, glomerular filtration rate as assessed by the estimated glomerular filtration rate (eGFR) ≥ 50 mL/min per 1.73 m2 calculated by the Modification of Diet in Renal Disease (MDRD) abbreviated formula, total bilirubin ≤1.5 x ULN, alanine aminotransferase (ALT) and aspartate aminotransferase (AST) ≤ 2.5 x ULN (≤ 5 x ULN for patients with liver involvement), alkaline phosphatase ≤2.5 x ULN (≤ 5.0 x ULN for patients with liver involvement and/or bone metastases).	
- Have serum lipase ≤1.5 x ULN.	
- Have adequate coagulation, as assessed by the following laboratory test results: International normalized ratio (INR) ≤ 1.5 or prothrombin time (PT) ≤ 1.5 x ULN, partial thromboplastin time (PTT) or activated PTT (aPTT) ≤ 1.5 x ULN.	
- Have a negative serum beta human chorionic gonadotropin (β-HCG) pregnancy test obtained within 7 days before the start of study treatment, in case of women with reproductive potential. For women of childbearing potential and men, agreement to use an adequate contraception for the duration of study participation and up to 4 months following completion of therapy for women and 6 months for male patients.	
- Must be able to comply with scheduled visits, treatment plan, laboratory tests and other study procedures.	
- Must be affiliated to a French health insurance

**Table 2:** Exclusion criteria

- Previous cancer within 5 years prior to study inclusion except for curatively treated cervical cancer in-situ, non-melanoma skin cancer and superficial bladder tumors (Ta, Tis and T1)	
- Diagnostic of metastases within 6 months after the termination of adjuvant chemotherapy.	
- Previous treatment for metastatic disease.	
- Active cardiac disease including any of the following: congestive heart failure (class 2 NYHA), new-onset angina (begun within the last 3 months), previous myocardial infarction (within the last 6 months), cardiac arrhythmias requiring anti-arrhythmic therapy (beta-blockers or digoxin are permitted).	
- ECG with a QT/QTc interval higher than 450 ms for men and higher than 470 ms for women.	
- Uncontrolled hypertension, i.e.,: systolic blood pressure > 140 mmHg or diastolic pressure > 90 mmHg despite optimal medical management	
- Arterial or venous thrombotic or embolic events such as cerebrovascular accident, deep vein thrombosis or pulmonary embolism within 6 months before the start of treatment.	
- Major surgical procedure, open biopsy, or significant traumatic injury within 28 days prior to first dose of treatment. Non-healing wound, ulcer, or bone fracture. History of gastrointestinal fistula or perforation.	
- Persistent NCI-CTCAE v5 gr.3 proteinuria, i.e.: urinary protein ≥3.5 g/24 h)	
- Peripheral neuropathy > gr.1	
- Ongoing infection > gr.2. Live attenuated vaccines are prohibited 10 days before the treatment, during the treatment and 3 months after the treatment	
- Known history of human immunodeficiency virus (HIV) infection and/or chronic hepatitis B or C infection.	
- Seizure disorder requiring medication.	
- Symptomatic metastatic brain or meningeal tumors.	
- Evidence or history of any bleeding diathesis, irrespective of severity. Any hemorrhage or bleeding event ≥ gr.3 within 4 weeks prior to the start of study medication.	
- History of organ allograft.	
- Dehydration ≥ gr.1.	
- Substance abuse, medical, psychological, or social conditions that may interfere with the patient’s participation in the study or evaluation of the study results.	
- Known hypersensitivity to any of the study drugs, study drug classes, or any constituent of the products.	
- Interstitial lung disease with ongoing signs and symptoms.	
- Concomitant intakes of St. John’s Wort.	
- Inability to swallow oral medication or any malabsorption condition.	
- Pregnant or breast-feeding subjects.	
- Participation in another clinical study with an investigational product during the last 30 days before inclusion. Any condition that, in the opinion of the investigator, would interfere with the evaluation of study treatment or interpretation of patient safety or study results	
- Patients who might be interconnected with or dependent on the sponsor site or the investigator.	
- Legal incapacity or limited legal capacity.	

### Biomarker studies and translational analyses

Serial blood samples are collected at several time points: at baseline before study treatment, every 2 cycles of treatment, and at treatment discontinuation. We monitor ccfDNA for efficacy or resistance of study treatment. We also plan to look within the available colorectal samples at the activity and expression of selected kinases been targeted by regorafenib.

### Ethics and regulatory considerations

The study was approved by French regulatory authorities (Agence nationale de sécurité du médicament et des produits de santé) on Jan 24th 2019, received a favorable opinion by the “Comité de Protection des Personnes EST III” (Dec 6th, 2018) and complies with the Helsinki declaration and French laws and regulations, and follows the International Conference on Harmonisation E6 (R2) Guideline for Good Clinical Practice. The trial results, even inconclusive, will be submitted in a peer-reviewed journal.

## Discussion

In fit patients with primarily unresectable mCRC, it becomes increasingly obvious to use frontline 3-CTx alone or combined with a targeted-agent, to get enough tumor shrinkage to allow conversion surgery or significant control of tumor burden. In this setting, RAS-mutated mCRC do not benefit the same from treatment than RAS-wild type mCRC do. Therefore, it appears reasonable to combine 3-CTx to the antiangiogenic agent bevacizumab in RAS-mutated mCRC, with the hope of improving outcome [2]. Unlike what it is known with bevacizumab, regorafenib exhibits not only antiangiogenic properties with cytostatic effects but also true cytotoxic effects as demonstrated in about 3% of heavily pretreated mCRC patients who presented with a tumor response in the CORRECT study [11, 15]. It is also worth noting that a clinical benefit favoring regorafenib over placebo was identified across *RAS* status [11, 12]. As preliminary studies have reported the safety and the efficacy of combining FOLFOX or FOLFIRI with regorafenib in mCRC [13, 14], it seems to us that it could be worth studying the combination of 3-CTx to regorafenib in *RAS*-mutant mCRC patients. On that line, we expect that this FOLFIRINOX-regorafenib combination will be able to challenge the FOLFIRINOX-bevacizumab regimen in primarily unresectable mCRC patients with *RAS*-mutant tumors.

We preferentially used FOLFIRINOX as backbone 3-CTx rather than FOLFOXIRI which was investigated by the GONO group [6] because we have a pioneered and long-lasting experience with FOLFIRINOX, both in the metastatic and in the adjuvant setting in colorectal and pancreatic cancer [4, 16–18]. The key difference between FOLFIRINOX and FOLFOXIRI relies on irinotecan and fluorouracil dosing (FOLFIRINOX: irinotecan 150 mg/m^2^, fluorouracil 2400 mg/m^2^ as a continuous infusion plus bolus fluorouracil 400 mg/m^2^; FOLFOXIRI: irinotecan 165 mg/m^2^, fluorouracil 3200 mg/m^2^ as a continuous infusion without any bolus of fluorouracil). As there is no evidence that FOLFIRINOX is different from FOLFOXIRI, we use the 3-CTx regimen we are confident with, i.e.: the FOLFIRINOX regimen. We used regorafenib continuation beyond the scheduled interruption of chemotherapy after 12 cycles. For patients who achieved disease control after 4 cycles of chemotherapy (most of the time 2-CTx with or without bevacizumab), the most common maintenance strategies are the following: maintenance with fluoropyrimidine or with fluoropyrimidine plus bevacizumab or observation [19]. Less is known for patients achieving disease control with 12 cycles of 3-CTx plus bevacizumab. In this clinical trial, the regorafenib use is all the more relevant for maintenance therapy that it provides (as opposed to bevacizumab monotherapy) an already demonstrated antitumour activity in mCRC [11, 12].

## Conclusion

The FOLFIRINOX-R study is the first phase I/II study to evaluate the safety and efficacy of regorafenib in combination with FOLFIRINOX as initial therapy for patients with primarily unresectable mCRC, which are found *RAS*-mutated on plasmatic ccfDNA assessment. Our objective is to demonstrate that this FOLFIRINOX-regorafenib combination provides enough clinical benefit to be tested in a controlled study in a selected patient population.

## Data Availability

Data sharing is not applicable to this article as no datasets were generated or analysed up to now for the current study.

## Abbreviations

CRC: Colorectal cancer
mCRC: Metastatic colorectal cancer
2-CTx: Chemotherapy doublet
3-CTx: Chemotherapy triplet
PFS: Progression-free survival
OS: Overall survival
ccfDNA: Circulating cell-free DNA
